# aPhyloGeo: a Python application for correlating genetic and climatic conditions

**DOI:** 10.1093/bioinformatics/btaf574

**Published:** 2025-10-21

**Authors:** Ana Laura Chenoweth Galaz, Nadia Tahiri

**Affiliations:** Department of Mathematics, University of Sonora, 83000, Sonora, Mexico; Department of Computer Science, University of Sherbrooke, QC J1K 2R1, Sherbrooke, Canada; Department of Computer Science, University of Sherbrooke, QC J1K 2R1, Sherbrooke, Canada

## Abstract

**Motivation:**

Environmental variation and its influence on genetic diversity is a central topic in evolutionary biology and phylogeography. Accurate correlations between genetic and climatic datasets to understand the genetic adaptations of different species to specific environments. It requires integrated and reproducible workflows.

**Results:**

We developed aPhyloGeo, an open-source and multiplatform application implemented in Python, for investigating correlations between genetic variation and environmental data within a phylogenetic framework. The workflow integrates multiple analytical steps, including sequence alignment, sliding window phylogenetic inference, and statistical approaches such as the Mantel test and the Procrustean randomization test. These analyses enable the identification of mutation hotspots that exhibit strong associations with environmental variables. In addition, aPhyloGeo supports multicore data processing and provides a fully reproducible pipeline for evaluating localized relationships between genomic variation and climatic distributions.

**Availability and implementation:**

aPhyloGeo is freely available on GitHub at: https://github.com/tahiri-lab/aPhyloGeo, as both a PyPI package and as Python scripts for Linux, macOS, and Windows.

## 1 Introduction

The relationship between genetic diversity and environmental heterogeneity is a central topic in evolutionary biology and landscape genetics. Phylogeography, defined as the study of the geographic distribution of genealogical lineages (Avise 2000), combines population genetics, spatial ecology, and biogeographic modeling to examine how historical and ecological factors shape diversification and connectivity (Knowles and Maddison 2007, Emerson and Hickerson 2008, Edwards *et al.* 2022). Reconstructing phylogeographic patterns requires several analytical steps. These include sequence alignment (Edgar 2004, Larkin *et al.* 2007, Katoh and Standley 2013), segmentation of genomic data using sliding windows (Li *et al.* 2025), computation of sequence similarities, and phylogenetic inference (Price *et al.* 2009, Nguyen *et al.* 2015, Kozlov *et al.* 2019), along with evaluation of genetic and ecological concordance with tests such as the Mantel test (Smouse *et al.* 1986) or PROcrustean randomization TEST (PROTEST) (Jackson 1995). In this context, tree similarity can be assessed using metrics such as the Robinson-Foulds distance (Robinson and Foulds 1981) and the Least Squares Distance (Steel 2016).

Although existing tools cover many of these tasks, available workflows are usually distributed across different programs and custom scripts, requiring manual integration of intermediate results. This complicates reproducibility, limits comparative analyses, and challenges researchers with limited computational training. Differences in input and output formats may introduce errors or inconsistencies, and a few software packages (Fares 2004, Kumar and Dudley 2007) provide efficient implementations of computationally demanding window-based methods (Raab *et al.* 2010). Consequently, studies linking genetic patterns with environmental gradients often rely on ad hoc solutions that are difficult to reproduce.

In this paper, we introduce *aPhyloGeo*, an open-source Python application that integrates genetic and environmental data for phylogeographic analysis. The package implements a comprehensive pipeline, including sequence alignment, sliding window segmentation, phylogenetic inference, evaluation, and ecological association testing. It provides preprocessing options for both sequence and climatic data and can incorporate diverse external data such as sequences, precomputed alignments, distance matrices, or phylogenetic trees. Its modular design allows researchers to adapt the workflow to specific datasets or questions without rewriting large parts of the pipeline, and standardized configuration files ensure that parameter choices are documented, supporting transparency and comparability across studies.

aPhyloGeo enables the study of localized genetic structure in relation to environmental gradients through phylogenetic trees and matrix-based statistical tests. Beyond practical applications, integrating genetic and ecological data within a single analytical framework allows the testing of hypotheses about the contributions of historical processes and environmental heterogeneity to observed genetic patterns. We describe its architecture, detail the genetic and climatic pipelines, outline preprocessing and analysis methods, and conclude with current limitations and potential directions for further development.

## 2 Materials and methods

### 2.1 Genetic pipeline—(green in Fig. 1)

#### 2.1.1 Sequence alignment

The genetic data workflow in aPhyloGeo uses raw nucleotide sequences in FASTA format. Users may provide pre-aligned sequences or perform alignments within aPhyloGeo. The internal alignment ensures positional homology required for downstream analyses, although it is more computationally demanding. The default procedure uses the Biopython pairwiseAligner module (v1.5.9), which implements dynamic programming algorithms such as Needleman-Wunsch and Smith-Waterman (Likic 2008) and is compatible with Biopython v1.79 (BSD 3-Clause License). To avoid licensing constraints associated with commonly used external tools [e.g. MAFFT (Katoh and Standley 2013), ClustalW (Larkin *et al.* 2007), MUSCLE (Edgar 2004)], aPhyloGeo provides alternative alignment functions that are ready to use without additional coding. Callable wrappers are included for users who prefer external aligners, with installation instructions detailed in the documentation. Aligned input files in standard formats may also be supplied directly to bypass the alignment step, reducing runtime when appropriate.

**Figure 1.**
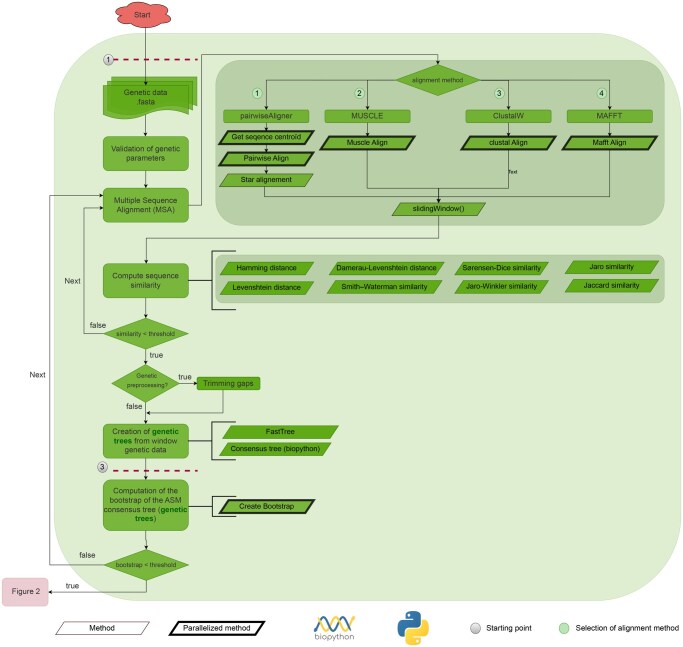
Workflow diagram representing the module (in green), which generates genetic phylogenies from sequence data using standard inference techniques. The workflow is optimized for parallelism and dynamic resource allocation to enable efficient processing across diverse computing environments.

#### 2.1.2 Sliding window

Once alignments are available, the sequences are partitioned into genomic windows by aPhyloGeo using a sliding window approach. Each window is analyzed separately, allowing the reconstruction of localized phylogenetic trees and the assessment of heterogeneity in evolutionary signals, such as those resulting from recombination, incomplete lineage sorting, or selection, across genomic regions (Giovanetti *et al.* 2022). Computations for each window can be accelerated using a multicore option, which distributes the analysis across available processor cores.

#### 2.1.3 Evaluation of sequence similarity

After alignment, sequence similarity is evaluated for each window using several established string similarity and distance metrics, including Hamming distance (Labib *et al.* 2019), Levenshtein distance (Berger *et al.* 2021), Damerau-Levenshtein distance (Zhao and Sahni 2019), Smith-Waterman similarity (Parvez *et al.* 2020), Jaccard similarity (Bag *et al.* 2019), Jaro and Jaro-Winkler similarity (Rozinek and Mareš 2024), and Sørensen-Dice similarity (Annathurai and Angamuthu 2022). These metrics provide complementary views of similarity, from strict character-based distances to set-based and probabilistic matching, and maintain the most variable alignments.

#### 2.1.4 Genetic data preprocessing

Prior to tree construction, alignments may undergo optional preprocessing to exclude low-quality regions. Specifically, columns exceeding a user-defined threshold are removed. This step reduces noise from poorly conserved or error-prone regions, improving the reliability of downstream phylogenetic inference (Comte *et al.* 2023).

#### 2.1.5 Tree construction

For each sequence alignment window, a bootstrap can be performed on that portion of the alignment; this allows the robustness of the sequences and the alignment to be evaluated. A tree is inferred from the bootstrap-resampled alignment using FastTree v2.1.11 (Price *et al.* 2010). For each window, a consensus tree is then constructed from the set of trees using the consensus module of Biopython v1.79 (BSD 3-Clause License).

#### 2.1.6 Bootstrap consensus

In order to evaluate the robustness of inferred phylogenies, a non-parametric bootstrap procedure (Felsenstein 1985) is implemented. It resamples alignment columns with replacement to generate replicate datasets. Each replicate produces a phylogenetic tree, and the frequency of clades across replicates provides an empirical measure of support. A majority-rule consensus tree is then constructed using Biopython v1.79 (BSD 3-Clause License) (Cock *et al.* 2009) by retaining clades present in >50% of replicates. Clades not meeting a user-defined support threshold are excluded from downstream comparisons.

### 2.2 Climatic pipeline—(blue in Fig. 2)

#### 2.2.1 Climatic data processing

Climatic datasets used in phylogeographic analyses typically include a broad set of environmental variables (e.g. temperature, precipitation, and solar radiation). To improve interpretability and reduce dimensionality, aPhyloGeo provides an optional variance-based filtering step that excludes variables below a user-defined threshold. This approach, based on the VarianceThreshold transformer from the scikit-learn library (Pedregosa *et al.* 2011), focuses analyses on the most informative environmental gradients while reducing computational complexity (Dormann *et al.* 2013).

**Figure 2.**
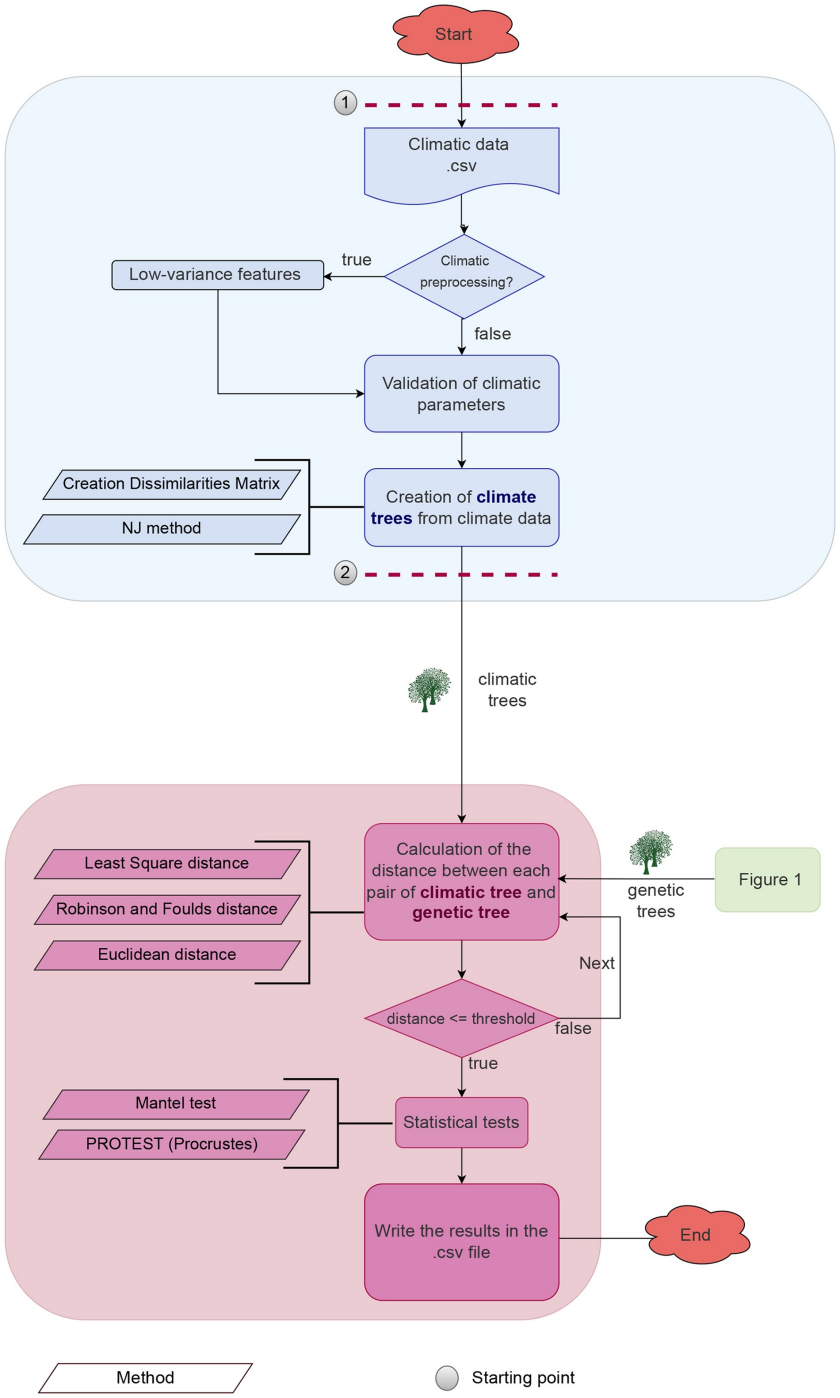
Workflow diagram representing a two-stage computational pipeline for integrative phylogeographic analysis. The first module (blue) constructs climate trees from environmental data. The second module (pink) compares climatic and genetic trees using multiple distance metrics, such as Robinson-Foulds, Least Squares, and Euclidean distance, to assess topological and spatial similarity. The outputs include, for each window (start and end positions), the climatic parameters, distances, and statistical values.

#### 2.2.2 Tree construction

After preprocessing, a dissimilarity matrix is computed from the retained variables using Euclidean distances between the geolocations of species. This matrix serves as input for tree reconstruction using the Neighbor-Joining (NJ) algorithm (Saitou and Nei 1987), a widely utilized distance-based method in ecological and phylogenetic studies. The resulting climatic tree represents environmental similarity across geographic locations and provides a reference for subsequent analysis with genetic phylogenies.

### 2.3 Phylogeography pipeline—(pink in Fig. 2)

#### 2.3.1 Distance measures between phylogenetic trees

aPhyloGeo supports several standard metrics for quantifying dissimilarity between phylogenetic trees. The Least Squares Distance (LSD) compares branch-length (patristic) distance matrices to capture both topological and branch length differences (Steel and Penny 1993). The Robinson-Foulds (RF) distance is a purely topological metric based on the number of discordant bipartitions between trees (Robinson and Foulds 1981). The Euclidean Distance (ED) can be applied to vector-based representations of trees, such as edge weights or clade frequencies, to measure dissimilarity in a continuous space (Danielsson 1980).

These additional metrics are used to select distance measures according to evolutionary signals and properties of the trees being compared.

#### 2.3.2 Statistical tests

To evaluate the relationship between genetic and climatic patterns, the Mantel test (Diniz-Filho *et al.* 2013) and the PROTEST (Quensen and Jackson 2023) are implemented. The Mantel test assesses the correlation between genetic and climatic distance matrices using a permutation-based approach. It utilizes average patristic distances from bootstrap consensus trees and Euclidean climatic distances among environmental variables. The PROTEST evaluates the correspondence between ordination configurations derived from genetic and climatic datasets, and assesses the statistical significance of this association. Both methods are implemented using Python-based statistical libraries (Sfiligoi *et al.* 2021) and allow configuration of parameters such as the number of permutations and the choice of correlation or alignment method.

## 3 Installation and documentation

All Python scripts for aPhyloGeo are publicly available without restriction at: https://github.com/tahiri-lab/aPhyloGeo and aPhyloGeo can be installed directly from the PyPI package at: https://pypi.org/project/aphylogeo/.

## 4 Conclusion and future work

In this work, we presented aPhyloGeo, an open-source, cross-platform Python application for phylogeographic analysis integrating genetic and environmental data. It performs sequence alignment, sliding window phylogenetic inference, consensus estimation, and statistical testing, enabling detection of localized genomic divergence and spatial correspondence between genetic and ecological variation in a reproducible pipeline.

Future work will focus on expanding similarity metrics (e.g. the geodesic in the Billera-Holmes-Vogtmann tree space or the Quartet distance), improving scalability through GPU acceleration and cloud deployment, supporting additional tree inference methods, and implementing a plugin system for community contributions. Future developments will also focus on extending the workflow to support additional input formats, including .nexus, to improve interoperability with a wider range of phylogenetic tools. Finally, in future work, we intend to extend the pipeline to include multivariate analyses, enabling the integration of several data types for a more thorough evaluation of correlations.
